# Molecular Phenotyping of AR Signaling for Predicting Targeted Therapy in Castration Resistant Prostate Cancer

**DOI:** 10.3389/fonc.2021.721659

**Published:** 2021-08-19

**Authors:** Agus Rizal A. H. Hamid, Maria V. Luna-Velez, Aleksandra M. Dudek, Cornelius F. J. Jansen, Frank Smit, Tilly W. Aalders, Gerald W. Verhaegh, Ewout Schaafsma, John P. M. Sedelaar, Jack A. Schalken

**Affiliations:** ^1^Department of Urology, Radboud University Medical Center, Nijmegen, Netherlands; ^2^Department of Urology, Ciptomangunkusumo Hospital, Faculty of Medicine, University of Indonesia, Jakarta, Indonesia; ^3^NovioGendix, Nijmegen, Netherlands; ^4^Department of Pathology, Radboud University Medical Center, Nijmegen, Netherlands

**Keywords:** castration-resistant prostate cancer, androgen receptor, gene amplification, splice variant, gene mutation, steroidogenic enzymes

## Abstract

Castration-resistant prostate cancer (CRPC) is defined by resistance of the tumor to androgen deprivation therapy (ADT). Several molecular changes, particularly in the AR signaling cascade, have been described that may explain ADT resistance. The variety of changes may also explain why the response to novel therapies varies between patients. Testing the specific molecular changes may be a major step towards personalized treatment of CRPC patients. The aim of our study was to evaluate the molecular changes in the AR signaling cascade in CRPC patients. We have developed and validated several methods which are easy to use, and require little tissue material, for exploring AR signaling pathway changes simultaneously. We found that the AR signaling pathway is still active in the majority of our CRPC patients, due to molecular changes in AR signaling components. There was heterogeneity in the molecular changes observed, but we could classify the patients into 4 major subgroups which are: AR mutation, AR amplification, active intratumoral steroidogenesis, and combination of AR amplification and active intratumoral steroidogenesis. We suggest characterizing the AR signaling pathway in CRPC patients before beginning any new treatment, and a recent fresh tissue sample from the prostate or a metastatic site should be obtained for the purpose of this characterization.

## Introduction

Castration-resistant prostate cancer (CRPC) arises when prostate cancer (PCa) progresses under castrate levels of serum testosterone which is achieved by androgen deprivation therapy (ADT) (1). CRPC is lethal disease and has poor prognosis. The treatment in CRPC ranges from immune therapy to targeted molecular therapy to cytotoxic chemotherapy. No single drug can be recommended to be given for treating CRPC (2). The PSA decline after the treatment of CRPC patients with novel drugs shows high variation (3–5). Until now, no method exists to identify in advance which CRPC patients may benefit most from targeting this pathway or which specific targets within the pathway deserve the most attention in an individual patient (6). Thus it calls for the need of a predictive biomarker which estimates the likelihood of response to a specific therapy. This will improve drug selection and tailor personalized therapy in CRPC. The benefits of personalized therapy have already been demonstrated in other tumor types, particularly in breast cancer (2, 7).

The development of predictive biomarkers needs to be based on the mechanism of disease. It has been shown that several escape mechanisms underlie the emergence of CRPC. These CRPC pathways can be classified into androgen receptor (AR)-dependent and AR-independent signaling pathways. Re-activation of the AR signaling pathway is still a major cause in CRPC. Aberrations in AR dependent signaling pathways still rely on AR-mediated transactivation, and include AR mutations, AR gene amplification, expression of AR splice variants (AR-V), intratumoral steroidogenesis and AR cofactor deregulation (6, 8, 9). This heterogeneity of aberrations in the AR signaling pathways may be responsible for the different and variable efficacies observed in clinical trials of novel agents, i.e., the binding AR mutation to antiandrogen may lead to reverse or agonist effect of antiandrogens (10).

However, previous studies have shown that each change in the AR signaling pathway, aberrant AR or increases in intratumoral steroidogenesis enzyme, accounted for only up to 30% cases, respectively (11, 12). Several AR splice variants (AR-V) displaying significant constitutive activity in the absence of ligand-binding have recently been reported, whilst the proportion of AR-V changes in patient were not described yet (13–16). The limitation of all previous studies is that in each study only one or two CRPC mechanisms are explored at the same time. There is no study that comprehensively explores multiple AR signaling changes in CRPC patients. The aim of our study is to evaluate comprehensively the molecular AR signaling phenotype inCRPC patients and propose a practical method to detect AR signaling changes as predictive biomarker for tailoring personalized therapy in CRPC.

## Material And Methods

### Patient and Tissue Selection

A total of 25 prostate tissue specimens from CRPC patients, which had obstruction symptoms, were collected with the approval of local ethical committee of the Radboud university medical center (RUMC). Prostate tissues were collected *via* transurethral resection of the prostate (TURP). We also used normal prostate (NP), benign prostatic hyperplasia (BPH) and primary prostate cancer without previous treatment as reference samples. These samples were obtained after radical prostatectomy for NP and PCa and TURP for BPH. The tissue specimens were snap frozen in liquid nitrogen or formalin-fixed and paraffin-embedded. Tissues containing at least 70% of tumor cells were selected after microscopic assessment of H&E-stained sections. Clinical data, such as age and previous treatment, were also collected (see **Table 1** and **Supplementary Table 2**).

**Table 1:** Patient characteristics.

**Age**	**median = 74 (59 – 83)**
**Treatment**:	**n**
Estrogen	1
Estrogen then change to orchiectomy	3
LHRH agonist	2
Antiandrogen	3
Orchiectomy	5
Orchiectomy + radiotherapy	1
Orchiectomy + Antiandrogen	5
LHRH agonist + antiandrogen	3
**Time to development of CRPC**	median = 3 years (1 – 8)

### Cell Culture

All prostate (cancer) cell lines were used as previously described (12). All cells were cultured in RPMI-1640 medium (Life Technologies), supplemented with L-Glutamine and 10% Fetal Bovine Serum (Sigma, F7524). Cells were maintained in a humidified atmosphere at 37°C and 5% CO_2_.

### Immunohistochemistry

Paraffin embedded tissue sections were used for immunohistochemistry. For AR staining, 5-um tissue sections were mounted and baked at 60°C for 1 hr. After deparaffination, antigen retrieval was performed in 10 mM sodium citric acid buffer (pH 6.0) at 95°C for 10 minutes. Non-specific binding was blocked by incubating the tissue sections with 5% human serum and 1% BSA in PBS for 30 minutes. Human AR protein was detected by using the AR N-terminus antibody (AR-N20, 1:5000, Santa-Cruz) and the AR C-terminus antibody (EP670Y, 1:200, Santa-Cruz). Primary antibody was incubated for 1 hour at room temperature. Poly HRP (Immunologic) secondary antibody was applied for 30 minutes at room temperature. DAB-H_2_O_2_ substrate (Immunologic) was added to the slides and incubated at room temperature for an additional 8 min. Tissue sections were counterstained slightly with hematoxylin. Slides were dehydrated and sealed with Permount Mounting Media for visualization by light microscopy. AKR1C3 data were used from a previous study (12).

### Genomic DNA and Total RNA Extraction

Genomic DNA was extracted using SDS/ProtK lysis (10 mM TRIS-HCl, 100 mM NaCl, 1% SDS, 1 mM EDTA and 2 mg/ml Proteinase K) for 1 hour at 65°C. Samples were purified by phenol and chloroform extraction. Then DNA was ethanol precipitated, and finally dissolved in DNAse-free water. Total RNA was extracted using TRIzol reagent (Life technologies), according to the manufacturer’s instructions. The concentrations of genomic DNA and total RNA were measured on a ND-1000 Nanodrop spectrophotometer (Thermo Scientific).

### AR Amplification

AR amplification was measured using a modified protocol from Ottesen et al. (17). Briefly, we used ~100 ng genomic DNA as a template for qPCR analysis, using primers specific for the *AR* gene and for the somatic single copy gene, glyceraldehyde-3-phosphate dehydrogenase (*GAPDH*) (**Supplementary Table 2**). SYBR Green qPCR was performed on a LC480 machine (Roche), with the following amplification conditions: 1 cycle at 95°C for 5 min, 45 cycles at 95°C for 10 s/60°C for 20 s/72°C for 20 s, followed by a melt-curve analysis. *AR* gene levels were normalized to the levels of *GAPDH*. To obtain AR copy numbers, the AR/GAPDH ratio was multiplied by 2. Ep156T cells (immortalized normal prostate epithelial cells; AR/GAPDH ratio = 1) and normal female lymphocytes (AR/GAPDH ratio = 2) were used as controls in every experiment.

### AR Mutation Analysis

Two micrograms of TRIzol-extracted RNA was DNase-I-treated and cDNA was synthesized using random hexamer primers and SuperScript II-MMLV reverse transcriptase (Life Technologies). The RT-reaction (30 µl) was diluted 4 times in H_2_O. Two µl of cDNA samples were used as a template. PCR was performed using AR exon-specific primers (**Supplementary Table 2**) on a MasterCycler machine (Eppendorf). Conditions for amplification were as follows 2 min. 95°C, followed by 36 cycles of 2 min. 94°C, 1 min 60°C, and 1 min 70°C. PCR products were evaluated by agarose gel electrophoresis, and then purified using the Wizard^®^ PCR Preps DNA Purification System (Promega). Purified PCR products were sequenced using AR exon-specific primers, the ABI PRISM BigDye Terminator Cycle Sequencing kit and an ABI PRISM 3730 automated DNA Analyzer (Applied Biosystems).

### Real-Time PCR

Gene expression was determined by SYBR Green qPCR, using SYBR Green PCR mix (Roche) and 2 µl cDNA as a template. RNA not subjected to reverse transcriptase was used as a negative control for PCR amplification. Gene-specific primers used are listed in **Supplementary Table 2**. Q-PCR was performed on a LightCycler LC480 instrument (Roche) (see *AR Amplification*). Crossing-point (Cp) values were determined using the LightCycler 480 SW 1.5 software (Roche). *HPRT* expression was used for normalization. Relative gene expression levels were calculated according to the model described by Pfaffl (18).

### Statistical Analysis

Comparison of AR protein levels with other patient characteristics were analyzed using Fisher’s exact test. The mRNA expression levels were analyzed using non parametric Mann-Whitney U test. Correlation of mRNA levels were analyzed using non parametric Spearman’s rho test. SPSS version 22 software was used for statistical analysis; p values < 0.05 were considered as statistically significant.

## Results

### Patient Characteristic

In this study, we evaluated 23 CRPC patients from whom fresh frozen and FFPE tissues were available for analysis. Various different treatments were used for advanced PCa before CRPC developed. Detailed patient characteristics are described in **Table 1**.

### AR Protein Expression

**Figure 1:**
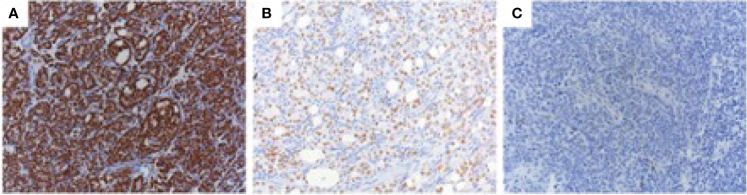
AR protein expression in CRPC. CRPC tissue specimens were stained with the AR N-terminal-specific antibody (N-20) using immunohistochemistry. Three different nuclear AR staining intensities were observed: strong **(A)**, intermediate **(B)**, and weak/negative **(C)**.

**Figure 2:**
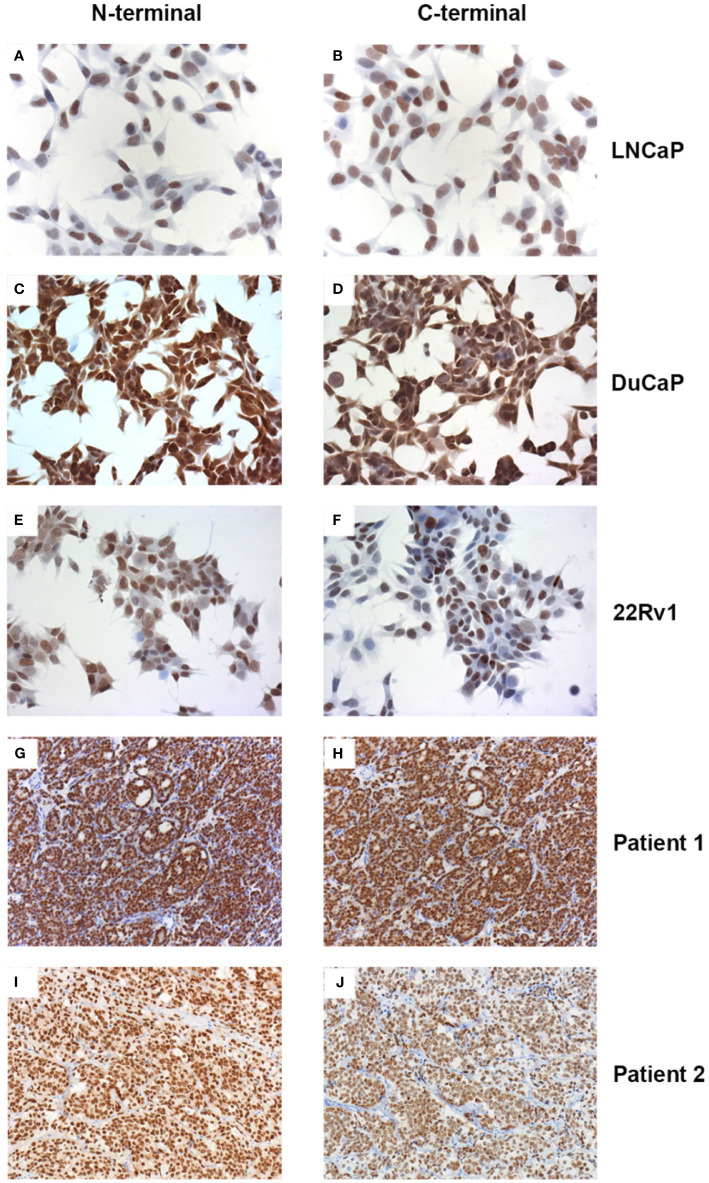
Expression of full length AR and variant AR proteins. Prostate cancer cell lines **(A–F)** and CRPC tissue sections **(G–J)** were stained with the AR N-terminus **(A, C, E, G, I)** or C-terminus **(B, D, F, H, J)** specific antibodies N-20 or EP670Y, respectively. For technical details see text.

We used an AR N-terminus-specific antibody to determine AR protein levels in patient samples. Most of the patients (20/23) had strong AR expressions, 4 patients had intermediate AR levels, whilst only 1 patient had low/no AR expression (**Table 2** and **Figure 1**). We also used an AR C-terminus-specific antibody for analysis, to specifically determine full length AR expression (16). We validated both AR N and C-terminus-specific antibodies in the LNCaP, DuCaP and 22Rv1 cell lines. It has been reported that LNCaP cells do not express AR splice variants, whilst DuCaP and 22Rv1 cells express high levels of AR-Vs that lack the C-terminus containing the AR ligand binding domain (LBD) (19, 20). Indeed, we found similar AR protein levels in LNCaP cells, using both the N and C-terminus-specific antibodies. Furthermore, using the C-terminus antibody, lower levels of AR protein were found in DuCaP and 22Rv1 cells (**Figures 2A** **–F**). We found that almost half of the CRPC samples, all showing high overall AR protein levels, showed reduced AR protein levels using the C-terminus-specific antibody (**Table 2** and **Figures 2G–J**), indicating that they express significant amounts of C-terminal truncated AR variants (that are only recognized by the N-terminus-specific antibody.

**Table 2:** AR and AKR1C3 IHC and AR amplification in CRPC.

AR protein level (based on N-terminal antibody)	n (%)
High	19 (83%)
Intermediate	3 (13%)
Low/no	1 (4%)
**AR protein level (N- versus C-terminal)**	
AR N = AR C	14 (61%)
AR N > AR C	9 (39%)
**AKR1C3 protein expression**	
High	6 (26%)
Intermediate	6 (26%)
Low/no	11 (48%)
**AR amplification status**	
Amplification	8 (35%)
Duplication	10 (44%)
Normal	5 (21%)

### AKR1C3 Expression

In a previous study, we determined AKR1C3 enzyme levels as a marker for active intratumoral steroidogenesis. High AKR1C3 expression was only observed in a subgroup of patients (~30%) (**Table 2**). We found a slightly higher, but not significant, proportion of high AKR1C3 expression in CRPC cases with high AR protein levels (**Supplementary Table 3**).

### *AR* Gene Amplification

To quantify *AR* gene amplification levels, the *AR* to *GAPDH* copy number ratio was determined by qPCR. We used DuCaP and VCaP cells, which have an amplified AR locus as positive controls (21). To determine a cut-off point for AR gene amplification, we used the AR/GAPDH ratio in the normal prostate cell line Ep156T, normal female lymphocytes, and BPH as reference points. The range of AR copy numbers in Ep156T and BPH were 0.52 – 1.81, and thus we used 2.0 as a cut-off point for AR duplications, and we used 4.0 as a cut-off point for AR amplification (22). We found that 8 out of 23 (35%) patients had AR amplification (**Table 2**). We found AR amplification was only in the high AR protein level group (**Supplementary Table 3**).

### AR Mutations

AR mutation analysis revealed that only one CRPC patient had a mutation (T868A) in the AR LBD, similar to the AR mutation in LNCaP cells. This patient had intermediate AR protein expression. We extended the AR mutation analysis to the entire AR gene (*i.e.* all AR exons) for patients that had received pure antiandrogen treatment (*i.e.* Bicalutamide, n=5). We also did not find any mutation in the non-LBD domains of the AR.

### AR Splice Variant (AR-V) mRNA Expression

**Figure 3:**
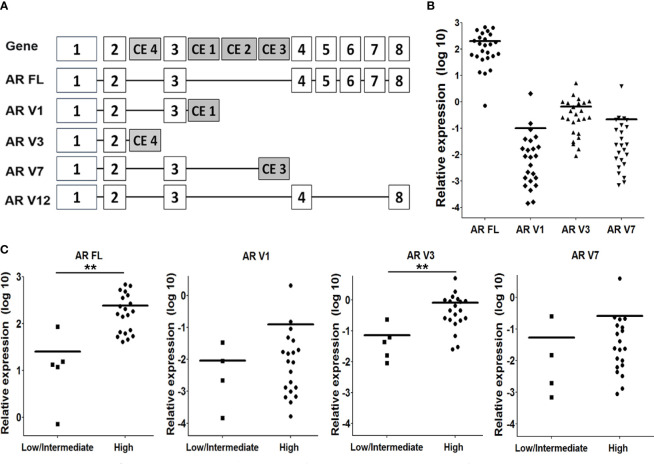
*AR* splice variants (AR-Vs) mRNA expression in CRPC tissue. **(A)** Schematic overview of the *AR* gene, and the structure of the full length (FL) AR and AR-V1, 3, 7 and 12. **(B)** Expression of AR FL and the AR-Vs was determined by qPCR analysis, using isoform-specific primers (see **Supplementary Table 1**), and using *HPRT* gene expression for normalization. **(C)** Comparison of AR FL and AR-Vs mRNA expression levels with nuclear AR protein staining intensities (as determined in **Figure 1**). Statistical analysis was performed using a non parametric Mann Whitney U test, **p < 0.05.

We selected to screen the mRNA expression levels of AR variants V1, V3, V7 and V12 (**Figure 3A**), because these variants have been described as the most abundant variant transcripts in prostate cancer (23). We could not detect V12 expression in our patient group. Full length AR and AR V1, V3 and V7 expression were significantly upregulated in CRPC cases, compared to primary PCa cases (**Supplementary Table 2**). However, the expression of AR-V transcripts did not exceed more than 1% of total AR (full length AR + AR-V) (**Figure 3B**). The expression of all AR-V isoforms was positively correlated with AR full length expression (**Supplementary Table 2**). Full length AR mRNA levels were also positively correlated with AR protein levels (Data not shown). We found that in patients with high AR protein levels, only AR-FL and AR-V3 mRNA levels were significantly increased (**Figure 3C**). There was no significant difference of AR FL and AR-V levels between the AR N = C and the AR N > C groups (data not shown).

## Discussion

The era of personalized cancer therapy has led to improved survival rates, reduced toxicity and increased efficiency in clinical trials through the selection of patients most likely to respond the therapy. However, the benefit of personalized therapy in CRPC is yet to be seen (24). The rational selection of appropriate populations of patients for molecular therapeutics is particularly important in a heterogeneous disease like CRPC (25). Thus, future directions in management of CRPC involve defining the activated signaling pathways in each patient to allow direct targeting of the altered pathways essential for tumor cell survival (26). Furthermore, with the increasing number of drugs available for CRPC, it is critical that the drug is now applied appropriately in order to maximize patient benefit (25). Until now, there is not yet a method to identify in advance which CRPC patients may benefit most from targeting a particular pathway or which specific targets within a pathway deserve most attention in an individual patient (27). In accordance to other studies, we found that the AR signaling pathway is still the most common escape mechanism in CRPC (8, 9). Most of our CRPC patient had high expression of AR protein.

Intratumoral steroidogenesis, one of the escape mechanisms in CRPC, has gained much attention in recent studies. High intratumoral androgen and steroidogenic enzyme levels were found indicative for active intratumoral steroidogenesis in CRPC (28). Until now, there is no consensus as to which biomarker can be used for detecting active intratumoral steroidogenesis. Many studies have shown AKR1C3 was specifically expressed at high levels in prostate tissue and in different metastases in a subgroup of CRPC patients (12, 29). Thus, we assumed that AKR1C3 can be used as a marker for active intratumoral steroidogenesis. Moreover, AKR1C3 is one of the potential targets for therapy in CRPC. Recently, a novel AKR1C3 inhibitor has been used in a clinical trial (30).

Amplification of the *AR* gene is one of the most frequent genetic alterations in CRPC. In congruence with previous studies, we found that approximately 30% of CRPC cases had high-levels of *AR* gene amplification (11). Until now there is no easy and simple clinical test for quantifying AR copy number changes (6). So far, AR amplification was evaluated using FISH analysis which is relatively time consuming. We adopted an AR qPCR technique, first described by Ottesen et al. (17), for the quantification of *AR* gene copy numbers. We validated this method for detecting AR amplification in DuCaP and VCaP cells which are known for demonstrating AR amplification. We suggested a cut-off point of > 4 as AR amplification similar with a cut-off point used in FISH analysis for high AR amplification (22). Advantages of this technique are that the results can be provided within a day and that the technique is easy and inexpensive (17).

To date, 159 *AR* mutations have been reported in PCa tissue (10). The prevalence of AR mutations in CRPC have varied from 10% up to 100% (27). This variation might be due to a variability in the analytical methodology used for the detection of AR mutations, highly selected patient tissue sampling, clinicohistopathological history and the inherent heterogeneity of CRPC (9). We sought to find AR mutations in the LBD because this domain is responsible for the binding of androgens. Mutations in the AR LBD result in promiscuous receptors that can also be activated by adrenal androgens, products of dihydrotestosterone metabolism, estrogenic and progestagenic steroids, and even by nonsteroidal antiandrogens, like hydroxyflutamide and nilutamide (10). Such mutations, therefore, may have a significant impact on anti-androgen therapy. However, we only found 1 AR mutation in our cohort of CRPC cases. The mutation found was identical to the one present in the LNCaP cell line.

Many studies have highlighted AR-Vs as key mediators of persistent AR signaling and resistance to the current ADT and next-generation AR directed therapies. We evaluated the expression of AR-Vs using N- and C-terminus-specific AR antibodies. Higher expressions of N-terminal compared to C-terminal ARs may indicate high AR-V expression. In this study, we found that half of the CRPC patients had higher N-terminal AR protein expressions than C-terminal AR protein expressions. However, we found that the relative expression of AR splicing variant transcripts was only 1% compared to full length AR mRNA expression levels. There was also no AR-V mRNA expression difference between the two N and C terminal groups (N=C and N>C). Even though, the half-life of AR-Vs is shorter than AR-FL, AR-Vs are more resistant to AR degradation process (31). Perhaps, the absence of androgens resulted in the higher levels of N- versus C-terminus containing AR. We have also found a correlation between increased AR-V and AR FL expression, supporting the notion that AR-Vs occur naturally as a consequence of aberrant transcriptional (elongation and termination) and splicing activity. The upregulation of AR in CRPC and the consequent increase in AR-V levels may exceed a critical threshold, leading to androgen-independent AR-V-mediated AR target gene activation (19).

**Figure 4:**
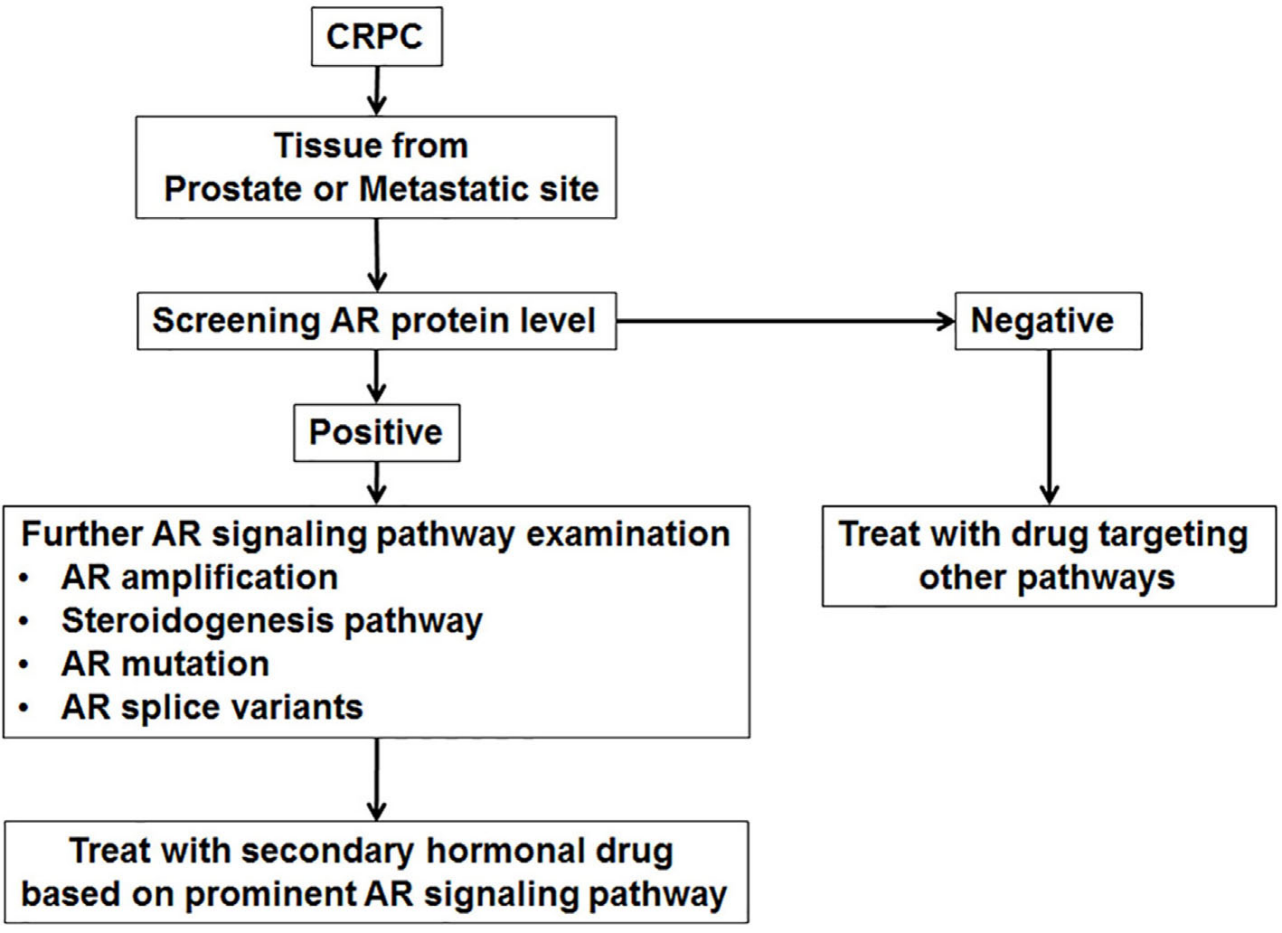
Algorithm of AR signaling pathways characterization in CRPC.

The aim of this study was to evaluate and validate several methods to explore simultaneously and comprehensively AR signaling pathway-related molecular changes, *i.e.* AR and AR-V expression, AR gene amplification and mutation, and AKR1C3 protein expression, in CRPC specimens. In our study, we found heterogeneity in the AR signaling pathway changes, and no single prominent alteration was found (**Figure 4**). This is the first study which explores AR signaling pathway changes comprehensively, using simple methods. These methods can be reproduced easily and can be performed fast in a clinical setting.

Due to the high frequency of (re-)activated AR signaling in our patients, we suggest performing an initial assessment of AR activity in CRPC patients before giving additional treatment (**Supplementary Figure 1**). Firstly, AR protein expression in CRPC patients is evaluated. Patients with no/null AR protein expression should not receive an AR targeted therapy, i.e. cytotoxic chemotherapy (docetaxel). *In vitro* studies have shown that AR null PCa cell proliferation is not influenced by (novel) AR targeting treatments (19, 32). Secondly, in AR protein positive cases, molecular AR abnormalities need to be investigated. Each AR aberration might influence the efficacy of AR targeted therapy. More potent antiandrogens are required to treat CRPC with AR amplification. However, it has already been shown that AR amplification is related to resistance to abiraterone or (novel) antiandrogens, such as enzalutamide (33). Patients with high AKR1C3 expression might have a good response to treatment with abiraterone acetate (CYP17A1 inhibitor) and/or would benefit from drugs targeting AKR1C3 (29). Patients with AR mutations and/or high AR-V levels most likely will not respond to drugs that target the LBD (C-terminal domain). Thus, novel antagonists that target the AR N-terminal domain are needed for the latter group of patients. Recently, such an N-terminus targeting drug has been discovered, but its clinical efficacy needs to be established in clinical trials (34).

The collection of CRPC tissue is one major drawback in CRPC personalized therapy. Fresh tissue needs to be obtained *via* biopsy of a recent tumor lesion in the prostate or at a metastatic site for each CRPC patient, and this may not be feasible for every patient for clinical or personal reasons (2).

The main limitation of this study is the small number of cases and samples analyzed. This is because we only included cases for which both fresh-frozen and FFPE tissue were available. Furthermore, for this exploratory study, we needed more tissue material to evaluate and validate several methods. Thus, we used CRPC specimens obtained by TURP which provided us with larger amounts of tissue. In the future, this study needs to be validated on biopsy-derived CRPC tissue. Additionally, the analyses were performed on primary tumors and the authors acknowledge that an evaluation of the actual AR signaling landscape is paramount to guide therapeutic choice.

We recommend future studies to explore the diagnostic value of AR levels in other tissues such as the lymph nodes, bones, or visceral metastases if available, with a varied range of clinical conditions such as less tissue samples or the use of biopsy cylinders. A larger study that compiles both clinical characteristics with histopathological findings is also warranted. The authors would also like to see future studies demonstrating the evolution of AR expression and alterations by comparing samples of different stages such as an initial diagnostic biopsy against a more advanced phase of CRPC. Future studies should also aim to assess the effect of previous different primary treatment i.e. external beam radiation therapy, on resistance to ADT. It is our hope that these additions to future studies could help drive proposals for therapeutic management based on AR signaling.

## Conclusions

We have developed and validated several methods to explore AR signaling abnormalities simultaneously in patient specimens. The AR signaling pathway is (re-) activated in the majority of CRPC patients. We suggest performing AR signaling profiling analysis before providing further treatment to CRPC patients.

## Data Availability Statement

The original contributions presented in the study are included in the article/**Supplementary Material**. Further inquiries can be directed to the corresponding author.

## Ethics Statement

The studies involving human participants were reviewed and approved by Local Ethical Committee of the Radboud University Medical Center (RUMC). The patients/participants provided their written informed consent to participate in this study.

## Author Contributions

All authors listed have made a substantial, direct, and intellectual contribution to the work, and approved it for publication.

## Funding

This work was supported by the Prostate Research Organizations-Network of Early Stage Training (PRO-NEST) project funded by the European Commission (FP7 Marie Curie ITN, contract #238278). The authors would also like to thank Universitas Indonesia for funding this research through Hibah Kolaborasi Riset Internasional Tahun 2019 grant with contract number NKB-1919/UN2.R3.1/HKP.05.00/2019.

## Conflict of Interest

The authors declare that the research was conducted in the absence of any commercial or financial relationships that could be construed as a potential conflict of interest.

## Publisher’s Note

All claims expressed in this article are solely those of the authors and do not necessarily represent those of their affiliated organizations, or those of the publisher, the editors and the reviewers. Any product that may be evaluated in this article, or claim that may be made by its manufacturer, is not guaranteed or endorsed by the publisher.

## Supplementary Material

Supplementary Table 1 Expression of AR splice variants in prostate cancer. **(A)** Comparison of AR FL and AR-Vs mRNA expression levels in primary PCa and CRPC tissue, using isoforms-specific qPCR. Statistical analysis was performed using non parametric Mann Whitney U test, **p < 0.05. **(B)** Correlation of AR FL and AR-Vs expression levels as determined by qPCR analysis. Linear regression analysis was performed to calculate the degree of correlation (r2 value). Statistical analysis was performed using the non-parametric Spearman’s rho test, **p < 0.05.
