# Supplementary material for: Molecular Phenotyping of AR Signaling for Predicting Targeted Therapy in Castration Resistant Prostate Cancer
Source: Front Oncol. 2021 Aug 19;11:721659. doi: 10.3389/fonc.2021.721659 (PMC8417043; doi:10.3389/fonc.2021.721659)
Supplement: Supplementary Table 1 — Expression of AR splice variants in prostate cancer. (A) Comparison of AR FL and AR-Vs mRNA expression levels in primary PCa and CRPC tissue, using isoforms-specific qPCR. Statistical analysis was performed using non parametric Mann Whitney U test, **p < 0.05. (B) Correlation of AR FL and AR-Vs expression levels as determined by qPCR analysis. Linear regression analysis was performed to calculate the degree of correlation (r2 value). Statistical analysis was performed using the non-parametric Spearman’s rho test, **p < 0.05. [file Table_1.docx]

**Suppl Table 1.** Detailed characteristic of CRPC patients

| Patient number | Age | Previous medication | Time to CRPC (year) | AR protein expression | AR amplification | AR N : C terminal ratio | AKR1C3 protein expression |
| --- | --- | --- | --- | --- | --- | --- | --- |
| 8 | 68 | Orchiectomy | 1 | High | Yes | N = C | High |
| 17 | 66 | Estrogen + Orchiectomy | 6 | High | Yes | N = C | Intermediate |
| 2 | 80 | Orchiectomy | 1 | High | Yes | N = C | No/Low |
| 10 | 68 | Orchiectomy + Radiotherapy | 2 | High | Yes | N = C | No/Low |
| 18 | 79 | Estrogen + Orchiectomy | 3 | High | Yes | N > C | Intermediate |
| 7 | 72 | Orchiectomy + Antiandrogen | 2 | High | Yes | N > C | No/Low |
| 12 | 74 | LHRH | 3 | High | Yes | N > C | No/Low |
| 19 | 82 | Estrogen + Orchiectomy | 2 | High | Yes | N = C | Intermediate |
| 1 | 76 | Estrogen | 4 | High | No | N = C | High |
| 6 | 59 | Orchiectomy + Antiandrogen | 2 | High | No | N = C | High |
| 11 | 60 | Orchiectomy + Antiandrogen | 2 | High | No | N = C | High |
| 15 | 77 | Antiandrogen | 5 | High | No | N = C | High |
| 5 | 78 | Orchiectomy | 8 | High | No | N = C | No/Low |
| 13 | 83 | LHRH | 6 | High | No | N > C | High |
| 9 | 82 | LHRH + Antiandrogen | 1 | High | No | N > C | Intermediate |
| 3 | 70 | Orchiectomy | 4 | High | No | N > C | No/Low |
| 14 | 76 | Antiandrogen | 4 | High | No | N > C | No/Low |
| 21 | 59 | LHRH + Antiandrogen | 1 | High | No | N > C | No/Low |
| 22 | 66 | Orchiectomy | 6 | High | No | N > C | No/Low |
| 20 | 81 | Antiandrogen | 1 | Intermediate | No | N = C | Intermediate |
| 23 | 70 | Orchiectomy + Antiandrogen | 3 | Intermediate | No | N = C | Intermediate |
| 16 | 76 | LHRH + Antiandrogen | 6 | Intermediate | No | N = C | No/Low |
| 4 | 69 | Orchiectomy + Antiandrogen | 1 | No | No | N = C | No/Low |

**Suppl Table 2.** Primers used for qPCR analysis

|  | Forward (5’ to 3’) | Reverse (5’ to 3’) |
| --- | --- | --- |
| AR (amplication) |  |  |
| AR exon 5 | CGACCAGATGGCTGTCATTC | CTGGAGTTGACATTGGTGAAGG |
| GAPDH | CTCCCCACACACATGCACTTA | TTGCCAAGTTGCCTGTCCTT |
| AR mutation | atgtcctggaagccattgag | atctgaaagggggcatgag |
| AR full length | aaggaactcgatcgtatcattgc | ttgggcacttgcacagagat |
| AR V1 | cgtcttcggaaatgttatgaagc | tgtggatgagcagctgagagtct |
| AR V3 (1/2/2b) | gtggaagctgcaaggtcttc | tttctGtcagtcccattggtg |
| AR V7 | cgtcttcggaaatgttatgaagc | gaatgaggcaagtcagcctttct |
| AR V12 (v567es) | ccaaggccttgcctgattgc | ttgggcacttgcacagagat |
| HPRT | CTCAACTTTAACTGGAAAGAATGTC | TCCTTTTCACCAGCAAGCT |

**Suppl. Table 3.** Correlation of AR protein levels with other characteristics

| **AR protein level** | **AKR1C3 expression** | |  |
| --- | --- | --- | --- |
|  | High | Low - Intermediate |  |
| High | 6 | 13 | p = 0,539 |
| Low - Intermediate | 0 | 4 |  |
|  |  |  |  |
|  | **AR amplification** | |  |
|  | Yes | No |  |
| High | 8 | 11 | p = 0,257 |
| Low - Intermediate | 0 | 4 |  |
|  |  |  |  |
|  | **AR N = AR C** | **AR N > AR C** |  |
| High | 10 | 9 | p = 0,127 |
| Low - Intermediate | 4 | 0 |  |
