# Supplementary figures and images for: Molecular Phenotyping of AR Signaling for Predicting Targeted Therapy in Castration Resistant Prostate Cancer
Source: Front Oncol. 2021 Aug 19;11:721659. doi: 10.3389/fonc.2021.721659 (PMC8417043; doi:10.3389/fonc.2021.721659)

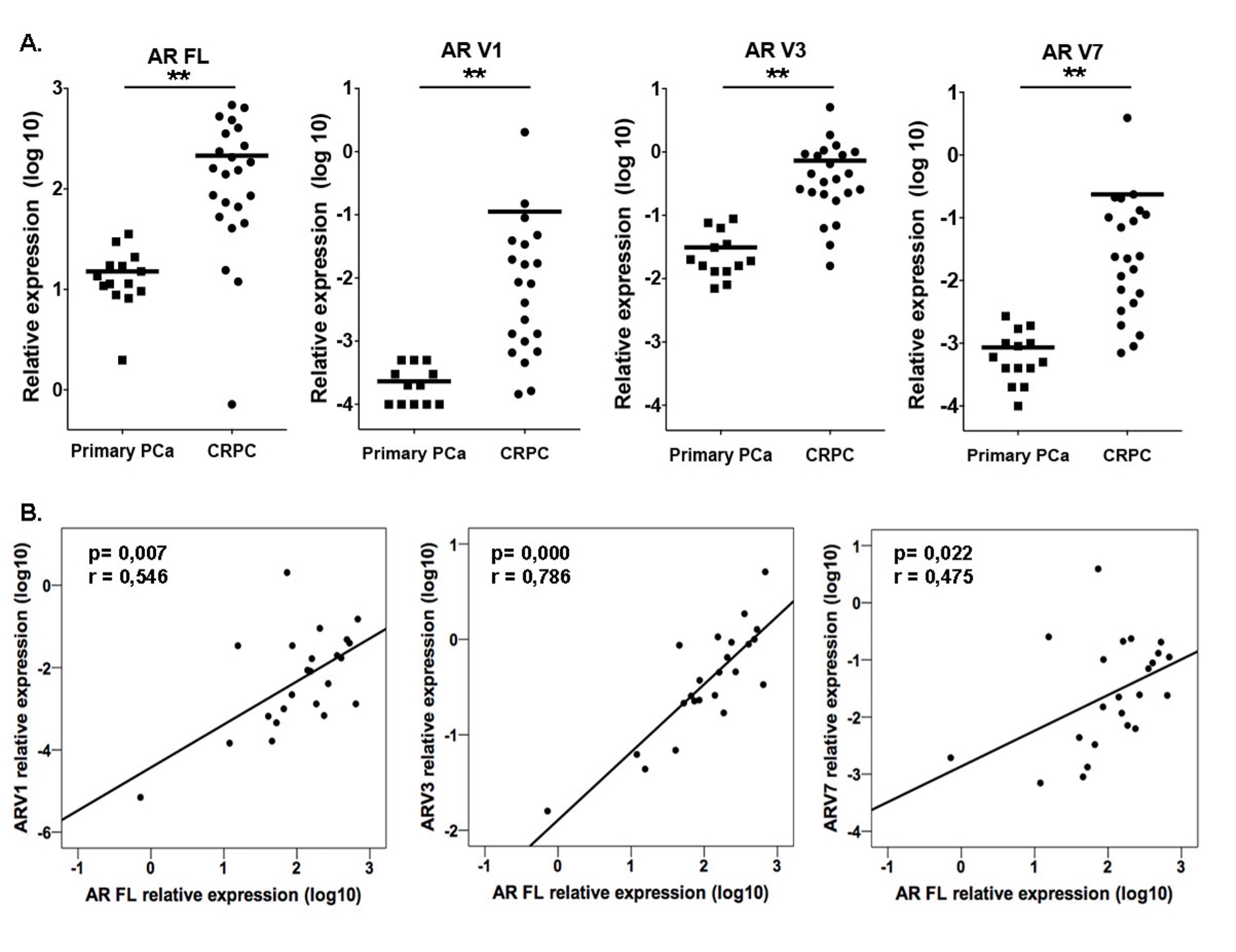

Supplement: Supplementary file 2 [file Image_1.jpeg]
